# Lead Break during Extraction: Predisposing Factors and Impact on Procedure Complexity and Outcome: Analysis of 3825 Procedures

**DOI:** 10.3390/jcm13082349

**Published:** 2024-04-18

**Authors:** Andrzej Kutarski, Wojciech Jacheć, Marek Czajkowski, Paweł Stefańczyk, Jarosław Kosior, Łukasz Tułecki, Dorota Nowosielecka

**Affiliations:** 1 Department of Cardiology, Medical University of Lublin, 20-059 Lublin, Poland; a_kutarski@yahoo.com; 2 2nd Department of Cardiology, Faculty of Medical Sciences in Zabrze, Medical University of Silesia, 40-055 Katowice, Poland; 3 Department of Cardiac Surgery, Medical University of Lublin, 20-059 Lublin, Poland; 4 Department of Cardiology, The Pope John Paul II Province Hospital of Zamosc, 22-400 Zamosc, Poland; 5 Department of Cardiology, Masovian Specialistic Hospital of Radom, 26-617 Radom, Poland; 6 Department of Cardiac Surgery, The Pope John Paul II Province Hospital of Zamosc, 22-400 Zamosc, Poland

**Keywords:** transvenous lead extraction, incomplete lead extraction, management of broken leads, complexity of extraction procedure

## Abstract

**Background**: Currently, there are no reports describing lead break (LB) during transvenous lead extraction (TLE). **Methods**: This study conducted a retrospective analysis of 3825 consecutive TLEs using mechanical sheaths. **Results**: Fracture of the lead, defined as LB, with a long lead fragment (LF) occurred in 2.48%, LB with a short LF in 1.20%, LB with the tip of the lead in 1.78%, and LB with loss of a free-floating LF in 0.57% of cases. In total, extractions with LB occurred in 6.04% of the cases studied. In cases in which the lead remnant comprises more than the tip only, there was a 50.31% chance of removing the lead fragment in its entirety and an 18.41% chance of significantly reducing its length (to less than 4 cm). Risk factors for LB are similar to those for major complications and increased procedure complexity, including long lead dwell time [OR = 1.018], a higher LV ejection fraction, multiple previous CIED-related procedures, and the extraction of passive fixation leads. The LECOM and LED scores also exhibit a high predictive value. All forms of LB were associated with increased procedure complexity and major complications (9.96 vs. 1.53%). There was no incidence of procedure-related death among such patients, and LB did not affect the survival statistics after TLE. **Conclusions**: LB during TLE occurs in 6.04% of procedures, and this predictable difficulty increases procedure complexity and the risk of major complications. Thus, the possibility of LB should be taken into account when planning the lead extraction strategy and its associated training.

## 1. Introduction

Transvenous lead extraction (TLE) plays a key role in lead management and is highly effective and safe, provided that all available safety measures are implemented [1,2,3]. Its goal is to remove all targeted leads in their entirety, with a minimal risk of any permanently disabling complication or procedure-related death [1,2,3]. Lead break (LB) during extraction is not a rare complication (approximated to 1.2–12%, with a simple average of 6.6%) [4,5,6,7,8,9,10,11,12,13,14,15,16,17,18], but only a few papers [19,20] and case reports describe how to manage broken leads [21,22,23]. Immediately after the rupture of the lead being extracted, only the tip of the lead may remain in the heart wall; a short (<4 cm) fragment may remain, with the tip in its original place; a long fragment (>4 cm) may remain, with the tip in its primary place; or a free-floating lead fragment (without connection to the heart wall and usually wandering within the blood stream) can be lost. There are surprisingly numerous case reports investigating the dislodgement of broken lead fragments into the pulmonary circulation, as well as the methods of their removal [24,25,26]. The broken lead fragment should be removed, if possible [1,2,3,7,18], because it can be a source of persistent or recurrent infection after a new system implantation [1,2,3,7,18]. Sometimes, the distal portion of the broken lead cannot be grasped with advanced tools, as it is surrounded by scar tissue. According to the current recommendations [1,2,3], abandonment of any fragment of the targeted lead precludes procedural success, but if the fragment is shorter than 4 cm (or it is just the tip of the lead), the procedure can be regarded as clinically successful in non-infectious cases.

To the best of our knowledge, no one has yet described in detail the scenario of lead break during extraction, the method and effectiveness of its management, or the impact of lead fracture on the level of TLE complexity and the occurrence of complications. Possessing a database of 3825 TLEs, we decided to investigate these problems.

### 1.1. Goal of the Study

The aim of this study was to evaluate the incidence of lead break during extraction, along with the method and effectiveness of its management, as well as the impact of this event on the occurrence of TLE complications and long-term patient survival.

### 1.2. Novel Elements

The goal of transvenous lead extraction is the removal of all targeted leads, without bodily harm and without procedure-related death. Regardless of the technique, there are situations in which fragments of broken leads remain in the cardiovascular system. This is referred to as the lack of procedural success (unless the cause was permanent bodily injury or procedure-related death). So far, no one has described the incidence of lead fracture during extraction, along with the method and effectiveness of its management, as well as its impact on the occurrence of extraction complications. To the best of our knowledge, this is the first report addressing this important problem.

## 2. Methods

### 2.1. Study Population

All transvenous lead extraction procedures were performed between March 2006 and February 2023. The study population consisted of 3825 patients (38.01% females) aged 5–99 years, with an average age of 66.04 years.

### 2.2. Lead Extraction Procedure

Indications for TLE, procedure effectiveness, and possible complications were defined according to the recent recommendations (2009 and 2017 HRS consensus and 2018 EHRA guidelines) [1,2,3].

#### 2.2.1. Procedure Complexity

Procedure complexity was expressed as the extraction time of all leads (sheath-to-sheath time) and the average time of single lead extraction (sheath-to-sheath/number of extracted leads), as well as indicating the use of second line and advanced tools [27,28,29,30].

#### 2.2.2. Unexpected Technical Problems during TLE

Unexpected technical problems were defined as situations which increased the level of procedural complexity but were not regarded as complications. They included blockage in the lead implant vein/subclavian region, preventing insertion of a polypropylene catheter in the subclavian vein; Byrd dilator collapse/fracture; lead-to-lead adhesion; the need to use an alternative approach; the loss of a broken lead fragment, when the main portion of the lead was dissected and removed, but both ends remained free; free-floating lead fragments, usually migrating into the pulmonary vascular bed; and the dislodgement of functional leads [27].

#### 2.2.3. Procedure Information

A standard stepwise approach, as previously described in Refs. [20,27], was used with all patients.

#### 2.2.4. Definition of Lead Break during Extraction

Lead break (LB) during extraction was defined as the fracture of all lead components, resulting in the formation of two separate parts (proximal and distal portions or proximal part and lead tip).

First, the fracture of the lead at the time of occurrence was classified as: 1. LB with a long (>4 cm) lead fragment; 2. LB with a short lead fragment (<4 cm); 3. LB with the tip of the lead only; 4. LB with loss of a free-floating lead fragment or lead tip. We invented this classification because all of the four types of lead breaks differ in regards to possible adverse clinical consequences, as well as the options and techniques used when trying to remove the remnants.

Second, the outcomes of the procedure (including broken lead management) were assessed as complete success (removal of the lead fragment in its entirety), partial success (reducing the length of the lead portion to <4 cm, or leaving in place an irremovable remnant < 4 cm, or leaving the lead tip only), or a lack of radiographic success (leaving an irremovable remnant > 4 cm, or leaving the entire lead, i.e., materials which were initially selected for removal).

#### 2.2.5. Extraction of Distal Fragments of the Broken Lead

We always tried to remove the broken lead, unless the broken remnant comprised just the tip of the lead. Depending on the location of the proximal end of the lead remnant, we attempted to grasp it with a lasso or basket catheter, using the implant vein access site, or using the subclavian approach, accessed by removing the other lead. Jugular or femoral approaches were used less frequently. After firmly grasping the proximal fragment of the broken lead, the lasso or basket catheter served as an extension of the broken lead, and we continued lead dilatation until the lead was removed. If it was unavailable, we recognized either partial radiographic success or the lack of radiographic success, resulting in no procedural success, depending on the length of the remaining lead [20,23]. Laser sheaths were not used.

Over the last 17 years, the extraction procedure has evolved from procedures performed in the electrophysiology laboratory, using intravenous analgesia/sedation, to procedures conducted in the hybrid room, employing general anesthesia [31].

### 2.3. Dataset and Statistical Methods

#### 2.3.1. Creation of Patient Subgroups for Analysis

The 3825 extraction procedures were divided into the following subgroups: 1. lead break with a long fragment (>4 cm): 95 procedures; 2. lead break with a short fragment (<4 cm): 46 procedures; 3. lead break including breakage of the tip of the lead only: 68 procedures; 4. lead break with loss of a free-floating fragment: 22 procedures; 5. all extractions with lead break (231, the sum of groups 1, 2, 3, and 4); 6. control group, all extractions without LB: 3594 procedures.

Subgroups 1–5 were compared with the control group to identify patient-, device- and procedure-dependent risk factors for lead break.

Next, we analyzed the outcomes of lead break management, defined as successful remnant removal; repeated fracture, with the lead fragment removed in its entirety; reduced length of the lead portion, with the retained fragment < 4 cm; failed remnant removal, with the retained fragment > 4 cm; unsuccessful attempt to grasp the fragment; no attempt made to grasp the fragment (no chances of success); lead remnant removal during emergency cardiac surgery; or procedure aborted due to major complications and ultimately, death of the patient.

#### 2.3.2. Statistical Analysis

All continuous variables are presented as means ± standard deviation. The categorical variables are presented as counts and percentages.

The significance of between-group differences was determined using the non-parametric Chi^2^ test, with the Yates correction, or the unpaired Mann–Whitney U test, as appropriate. Univariable and multivariable linear regression analysis was used to determine the predictors of LB during extraction. The variables achieving *p* < 0.05 were entered into the multivariable model. Two multivariable models were built: one for clinical and CIED-related variables and one for risk scores of TLE (procedure complexity or occurrence of major complications). The Kaplan–Meyer survival curves were plotted for the patients divided into groups, depending on the type of the broken lead. Differences between curves were tested using the log-rank test. A *p*-value less than 0.05 was considered as statistically significant. Statistical analysis was performed using Statistica 13.3 (TIBCO Software Inc., Tulsa, OK, USA).

### 2.4. Approval of the Bioethics Committee

All patients gave their informed written consent to undergo TLE and to use anonymous data from their medical records, as approved by the Bioethics Committee at the Regional Chamber of Physicians in Lublin no. 288/2018/KB/VII. The study was carried out in accordance with the ethical standards of the 1964 Declaration of Helsinki.

## 3. Results

As for the potential patient-related risk factors, younger patient age at first CIED implantation and during TLE, a higher ejection fraction, and a lower Charlson co-morbidity index were significant predictors of lead break during extraction. However, patient gender, type of underlying heart disease, and degree of heart failure had no association with the fracture of the removed lead. It can be assumed that lead break during removal was not related to the type of indications for TLE, except in the case of mechanical lead failure, for which there may be some link (Table 1).

The risk factors for increased procedure complexity [27], i.e., implant duration, abandoned leads, unnecessary (large) lead loops in the heart, the number of leads before TLE, leads on both sides of the chest, and the number of CIED-related procedures before lead extraction, were found to have a significant impact on the chance of breaking the targeted lead, and therefore, these can be considered as the risk factors for lead break during extraction.

The middle panel of Table 2 summarizes the risk scores for the identification of major complications or increased procedure complexity and their value in predicting lead break during extraction. Higher SAFeTY scores [32], MB scores [29], and LED index [28] indicated an increased risk of lead break, but the recently described LECOM score [27] appeared to be the most valuable indicator. Calculators such as the EROS score [33] and the Advanced TLE score [30] were of little value in predicting the risk of lead break.

The lower panel of Table 2 shows potential procedure-related risk factors for major complications and increased procedure complexity, indicating that the number of extracted leads per patient, the extraction of abandoned leads, the extraction of unipolar leads, passive fixation leads, leads with abnormal loop in the heart, and leads with long dwell times are also procedure-dependent risk factors for lead break during extraction.

We compared procedure complexity in patients with lead break and in the control group and found that all indicators of increased procedure complexity, such as lead extraction time and overall number of unexpected procedure difficulties, referred to as technical problems, were significantly higher in patients with LB during extraction. The need to use additional tools (Evolution (old and new) or TightRail, metal sheaths, lasso catheters/snares, basket catheters) and techniques (need to change or use an additional venous approach) was also significantly more common in patients with LB during extraction. All indicators were included simultaneously in the retrospective TLE combined difficulty score (lead dilatation time and the use of second-line tools, advanced tools, and advanced techniques). The values of this score were significantly higher in patients with lead break (Table 3).

In summary, each type of lead break (with a long or short lead remnant, of break of only the tip of the lead, or free-floating lead fragments) is associated with a high level of procedure complexity, which is not always due to attempted remnant removal, as is the case with leaving in place the tip of the lead.

**Figure 1:**
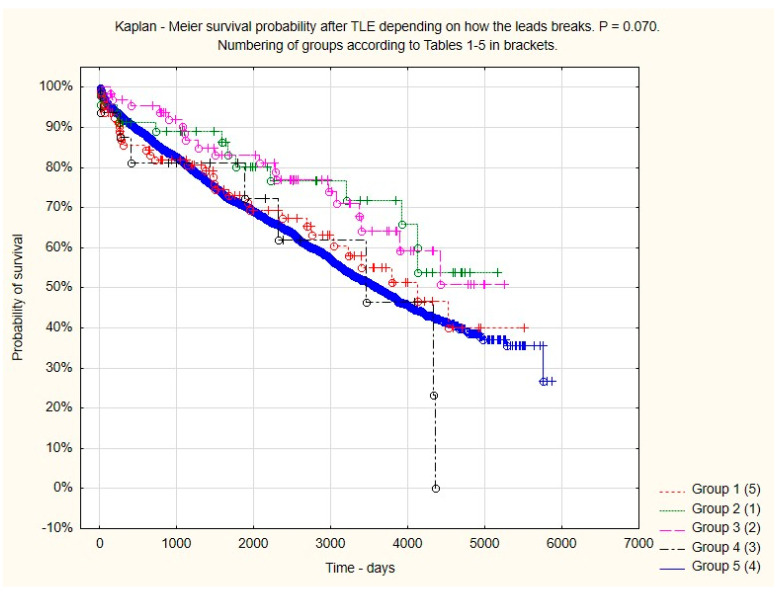
Long-term survival in patients with various types of lead break during extraction and in patients in the control group.

TLE-related major difficulties, such as any major complication, hemopericardium, severe tricuspid valve damage, and the need for rescue cardiac surgery, were significantly more frequent in patients with different forms of lead break (except for a rare complication, such as a hemothorax). However, despite the complications, there was no procedure-related death (intra-, post-procedural) in the LB subgroups. On the other hand, the need to leave in place non-removable lead fragments (especially in patients with infections), and more frequent damage to the tricuspid valve resulted in a significantly lower percentage of clinical and procedural successes. However, on the other hand, the one-year patient mortality and that for the entire follow-up period were similar in all the subgroups as compared to that of the control group. Therefore, it can be assumed that the mere fact of LB does not impact the length of survival after TLE (Table 4, Figure 1). 

Table 5 presents the management of LB and its effectiveness in five patient subgroups. Long lead fragments (>4 cm) were removed entirely in 66.32% of cases, the remnant length was reduced to less than 4 cm in 27.37% of cases (due to subsequent break of the lead), and the attempted retrieval of lead fragments was unsuccessful in only 3.16% of cases; thus, the remnant was left in place. Short lead remnants (<4 cm) were successfully grasped and removed in 8.70% of cases, and their length was reduced in 2.17% of cases. An attempt to grasp the lead fragment was unsuccessful in 21.74% of cases, and in most cases (63.04%), there was no chance to grasp it, and no attempt was made to retrieve the fragment.

In all cases of loss of a free-floating lead fragment, an attempt to retrieve the lost fragment was made. It was most frequently grasped in the pulmonary vein, rarely in the brachiocephalic vein or superior vena cava, and even in the liver vein. Retrieval was successful in 68.18% and only partially successful in 13.64% of cases.

**Figure 2:**
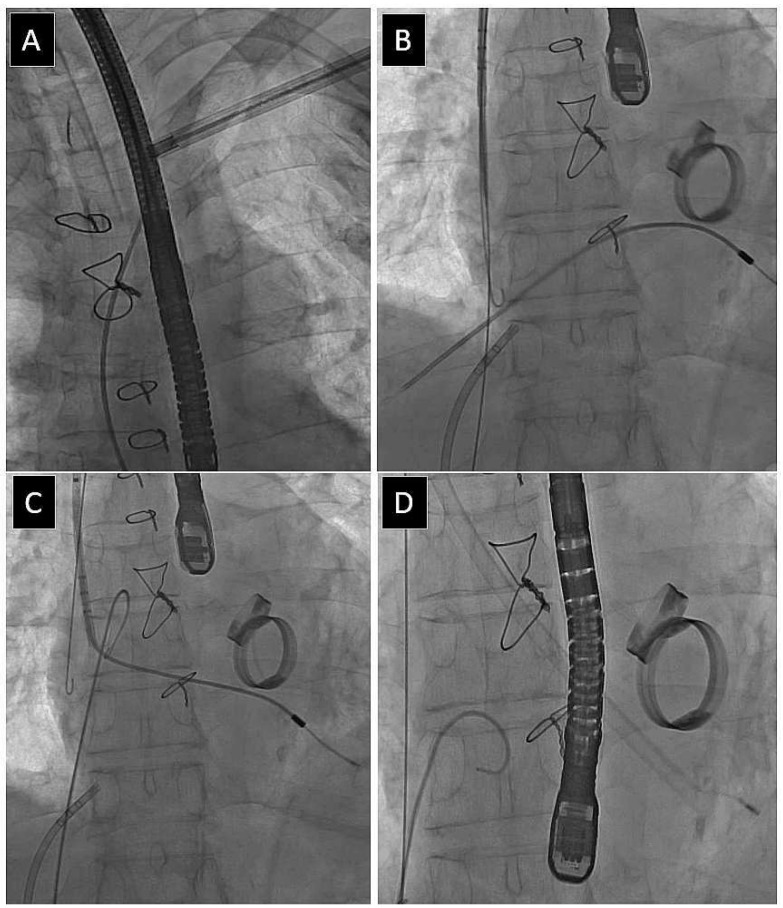
Extraction of an old model passive fixation lead. Due to lack of progress in lead dilatation, an Evolution sheath was used (**A**). Unfortunately, the lead was broken, but it was possible to dissect its proximal end from scar tissue using a curved catheter and an angiographic guide wire (**B**). In the next stage, the proximal end of the lead fragment was grasped with a lasso catheter, working inside the CS lead implantation sheath (**C**). Lead dilatation was continued using a larger polypropylene sheath (**D**).

**Figure 3:**
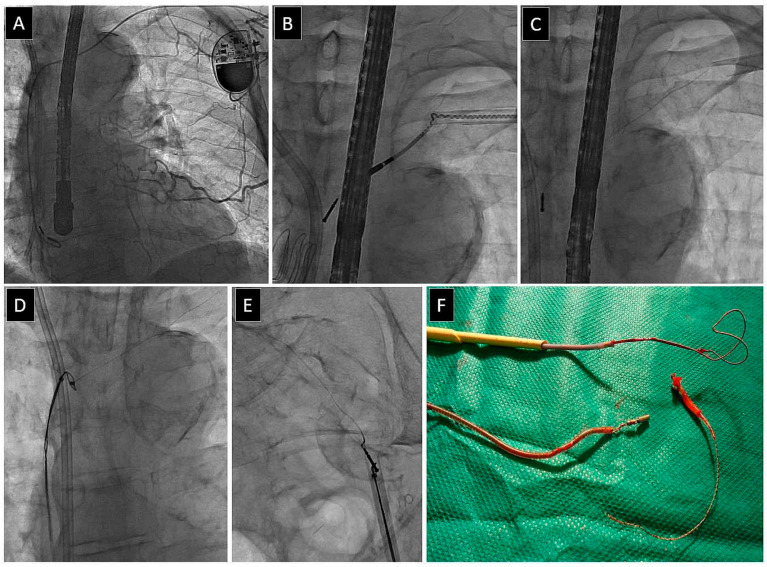
VVI pacing system (L) and a permanent hemodialysis catheter (R). Severe venous obstruction (**A**). Subclavian crush syndrome—trying hard to pass through the lead venous entry. Partial extraction of the targeted lead and signs of its destruction (**B**). Disaster—break of the lead being extracted; the tip, with the internal conductor, remains. Small chances of successful removal and high risk of blockage in the subclavian vein (**C**). Successful grasping of the lead fragment via the femoral approach and removal via the femoral vein (**D**,**E**). To avoid vein damage, yellow polypropylene sheaths were used to cover the extracted and grasped lead fragment (**C**,**F**).

**Figure 4:**
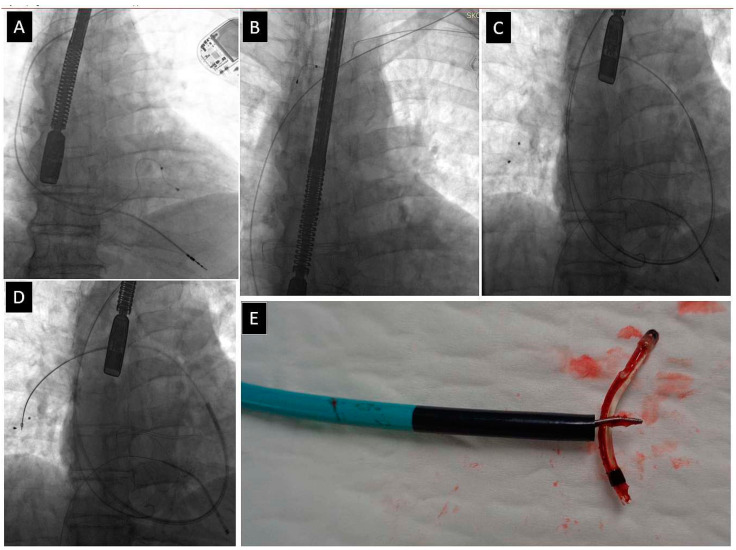
BiV (CRT-P without a lead) pacing system—infection. (**A**) Right ventricular lead extracted (temporary guide wire remained). Problem with the passage of a thin sheath via the brachiocephalic vein. Unintentional extraction of the lead (**B**). Break of the distal part of the lead, which escaped quickly, with blood, into the pulmonary circulation (right inferior pulmonary artery) (**C**). Passage to the pulmonary trunk using a catheter intended for implantation of a left ventricular lead, and then insertion of a lasso catheter into the right inferior pulmonary artery to grasp the remnant (**C**,**D**). Remnant removed (**E**).

**Figure 5:**
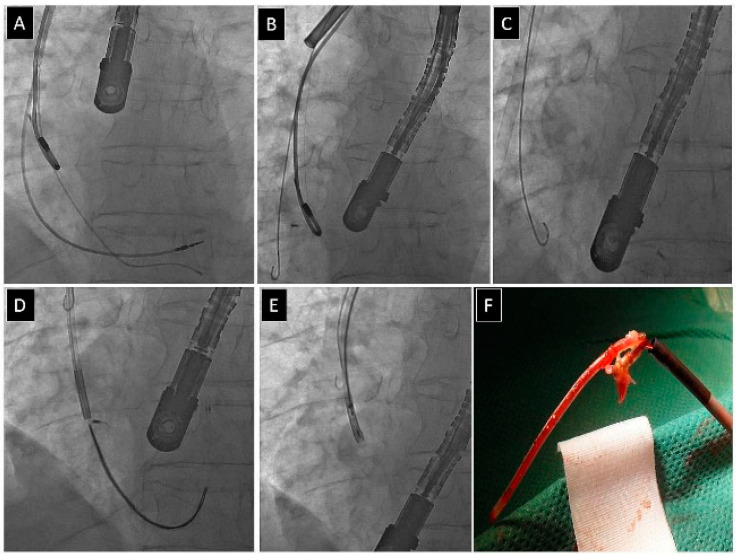
Removal of a DDD pacing system with an abandoned unipolar lead (**A**). Unipolar lead stretching, little progress despite using a mechanical rotational (TightRail) sheath (**B**). Bipolar atrial lead was extracted. Attempted extraction using a polypropylene catheter resulted in fracture of the lead, with dissection of its tip (**C**), which detached from the lead and “danced” to the right ventricle first (**C**) and then to the right atrium (**D**). A dozen or so openings and closings of the lasso catheter made it possible to grasp the “tip” of the lead and hide it in the “mother” catheter (Attain CS sheath) (**E**). X-ray image does not always reflect the size of the remnant. A fragment of the silicone tube made it possible to grasp the “tip” of the lead (**F**).

Figure 2, Figure 3, Figure 4 and Figure 5 present examples of different options for broken lead management: removal of the broken long lead fragment using a combined approach, with removed fragment dilatation (Figure 2), removal of the broken long lead fragment from the left brachiocephalic vein using the femoral approach (Figure 3), removal of the broken long lead fragment from a branch of the pulmonary artery using extracted lead venous access (Figure 4), and removal of the broken free-floating short lead fragment from the right atrial space using the superior approach) (Figure 5).

In summary, it can be stated that in most cases of lead break during extraction, it is possible to remove the lead remnant in its entirety; in a certain percentage of cases, as a result of subsequent breaks, the size of the remnant can be reduced (<4 cm); and rarely, the remnant remains irretrievable. Thus, lead break has a greater impact on procedure complexity than on the lack of complete radiographic success.

As was shown in Table 6, a larger number of lead dwell times [OR = 1.018, *p* = 0.006], a higher left ventricular ejection fraction [OR = 1.014, *p* = 0.013], a larger number of previous CIED-related procedures [OR = 1.187, *p* = 0.016], and the extraction of passive fixation leads [OR = 6.354, *p* < 0.001] were the predictors of LB during extraction. Older patient age at first system implantation decreased the risk of lead break during extraction [OR = 0.971, *p* < 0.001]. 

Multivariable regression analysis revealed that LECOM [OR = 1.019, *p* < 0.001] and LED scales [OR = 1.058, *p* < 0.001] (both used for prediction of TLE complexity) had the highest predictive value (Table 7).

## 4. Results Summary

Lead break during TLE occurs in approximately 6% of TLE procedures and appears to be a predictable event. A larger sum of lead dwell times, a higher left ventricular ejection fraction, a larger number of previous CIED-related procedures, and the extraction of passive fixation leads were the strongest predictors of lead break during TLE. Some scores (LED index and LECOM score) can predict an increased risk of lead break.

All forms of lead break are associated with increased procedure complexity, expressed as lead extraction time and number of unexpected procedure difficulties (technical problems), as well as the necessity of using additional tools and advanced techniques. Patients with lead breaks were significantly more likely to have major complications of TLE; nevertheless, there were no procedure-related deaths (intra-, post-procedural) in these patients, and LB did not affect the length of survival after TLE.

In most patients with lead break during extraction, it is possible to remove the lead remnant in its entirety; in a certain percentage of cases, as a result of subsequent breaks, the size of the remnant can be reduced to <4 cm; and rarely, the remnant remains irretrievable. Thus, lead break has a greater impact on the complexity of the procedure than on the lack of complete radiographic success.

## 5. Discussion

Many reports on TLE effectiveness do not provide data on the incidence of lead fragments left in place. The investigators provide rates of procedural success (lead removal without partial residue of lead materials, in the absence of permanently disabling complication or procedure-related death) and rates of procedure-related deaths. Taking into account the small percentage of tricuspid valve damage, stroke, etc., the rate of incomplete lead removal can be approximated to 1.2–12%, with the simple average being 6.6%. The rate of no radiographic success does not seem to depend on the use of first-line tools. For conventional techniques using non-powered polypropylene sheaths, the rate is 12–13%, and the simple average is 6.4% [4,5,6,7,8]; for laser treatment, the rate is 4–11%, with a simple average of 7.0% [9,10,11,12,13]; for mechanical rotational sheaths with a manual drive (Evolution, TightRail), the rate is 4–7%, with a simple average of 5.5% [14,15]; and for the femoral approach technique, the rate is 7–11%, and the simple average 9.0% [16,17].

During the analysis of infectious patients, incomplete lead removal was considered as a risk factor for mortality at a later time. Similarly, incomplete lead removal in infectious patients is considered as a factor precluding clinical success, although the interpretation of this phenomenon differs slightly between the HRS 2017 and EHRA 2018 recommendations [1,2,3,18]. To the best of our knowledge, no one has analyzed lead break and its risk factors in detail, nor described the outcomes of remnant removal.

In the literature, we found only one report of lead damage evealuation, by Morita et al., who described 11 cases of lead damage during lead extraction (“lead break during removal was defined as the lead stretching and becoming misshapen, as assessed by fluoroscopy”), but without breaking the continuity of the metal conductor [34]. It is obvious, however, that in the case of the extraction of leads with a shorter stay in the patient’s body, the procedure is likely to be of low complexity, the risk of major complications is negligible, and selected patients even have a chance of being discharged on the same day [35].

Our analysis of 3825 extraction procedures showed that during TLE, using conventional and rotational mechanical sheaths, the rate of lead break (any form) is as follows: LB with a long fragment, 2.48%; LB with a short fragment, 1.20%; LB with a break of the tip of the lead only, 1.78%; and LB with loss of free broken lead fragments, 0.57%; the rate of all extractions with LB was 6.04%, which is very similar to that quoted in the literature [4,5,6,7,8,9,10,11,12,13,14,15,16,17,18]. But we proved that in patients in whom a lead remnant is equivalent to a lead fragment (more than the tip only), there is a 50.31% chance of removing the remnant in its entirety and an 18.41% chance of significantly reducing its length (to less than 4 cm). It appears that the management of lead break and the removal of broken lead fragments should be part of the training in regards to transvenous lead extraction.

## 6. Conclusions

1. Lead break during lead extraction occurs in 6.04% of cases, but broken lead fragments can be removed in 35.50% of patients, or the length of these fragments can be significantly reduced in 12.99% of patients.
2. A larger sum of lead dwell times, a higher left ventricular ejection fraction, a higher number of previous CIED-related procedures, and the extraction of passive fixation leads were the strongest predictors of lead break during extraction. Some scores (LED index and LECOM score) predicted an increased risk of lead break.
3. Lead break increases procedure difficulty and complexity, as well as the risk of TLE-related major complications. Thus, the possibility of lead break should be taken into account when planning the removal strategy and training in regards to transvenous lead extraction.

## 7. Study Limitations

The study has several limitations. It is a three-center study, but the procedures were conducted by of the same first operator. All procedures were performed using various types of mechanical systems, with the exception of laser-powered sheaths. We have not used laser energy catheters in the past, for economic reasons, but also because, with mechanical rotation sheaths at our disposal, we did not feel the need to use more aggressive tools. The main weakness of this study was the use of a unique technique of grasping and removing lead remnants using a re-established lead venous entry approach, devised and used for 17 years, which made it possible to obtain such results. 

## Figures and Tables

**Table 1:** Potential patient-related risk factors: characteristics of patient groups and main indications for lead extraction.

Patient-Related Predictors of TLE Complexity, Major Complications and Coexisting Indications for TLE	Lead Break with a Long Fragment	Lead Break with a Short Fragment	Lead Break of the Tip of the Lead Only	Lead Break with Loss of a Free-Floating Fragment	All Extractions with Lead Break	All Extractions without Lead Break
	Mean ± SD N (%) M–W U test, Chi^2^ test	Mean ± SD N (%) M–W U test, Chi^2^ test	Mean ± SD N (%) M–W U test, Chi^2^ test	Mean ± SD N (%) M–W U test, Chi^2^ test	Mean ± SD N (%) M–W U test, Chi^2^ test	Mean ± SD N (%)
Group number/number of patients (%)	1/95 (2.48)	2/46 (1.20)	3/68 (1.78)	4/22 (0.57)	5/231 (6.04)	6/3594 (93.96)
Patient age during TLE (years)	59.29 ± 19.36 *p* < 0.001	56.67 ± 21.57 *p* = 0.001	60.40 ± 19.79 *p* = 0.022	66.09 ± 14.03 *p* = 0.724	59.75 ± 19.55 *p* < 0.001	66.44 ± 15.34
Patient age at first system implantation (years)	43.65 ± 20.39 *p* < 0.001	41.93 ± 22.28 *p* < 0.001	47.74 ± 21.35 *p* < 0.001	50.14 ± 17.71 *p* = 0.015	45.14 ± 20.87 *p* < 0.001	58.14 ± 15.34
Female	48 (50.53) *p* = 0.016	17 (36.96) *p* = 0.970	25 (36.76) *p* = 0.964	6 (27.27) *p* = 0.427	96 (41.56) *p* = 0.282	1358 (37.79)
Ischemic heart disease	31 (32.63) *p* < 0.001	18 (39.13) *p* < 0.001	37 (54.41) *p* = 785	11 (50.00) *p* = 0.669	97 (41.99) *p* < 0.001	2042 (56.82)
NYHA FC III or IV	12 (12.63) *p* = 0.491	4 (8.70) *p* = 0.268	11 (16.18) *p* = 0.938	4 (18.18) *p* < 0.830	34 (14.72) *p* = 0.738	567 (15.78)
LVEF (%)	55.14 ± 11.98 *p* < 0.001	55.41 ± 10.58 *p* = 0.003	54.04 ± 12.53 *p* = 0.015	52.59 ± 13.49 *p* = 0.266	54.63 ± 11.98 *p* < 0.001	49.14 ± 15.44
Charlson co-morbidity index (points)	4.12 ± 4.14 *p* < 0.001	3.04 ± 3.11 *p* < 0.001	3.53 ± 3.15 *p* = 0.003	3.96 ± 3.15 *p* = 0.421	3.71 ± 3.59 *p* < 0.001	4.81 ± 3.69
Indications for TLE						
Infective endocarditis, with or without pocket infection	14 (14.74) *p* = 0.125	12 (26.09) *p* = 0.608	14 (20.59) *p* = 0.978	7 (31.82) *p* = 0.385	47 (20.35) *p* = 0.651	785 (21.84)
Local (isolated) pocket infection	11 (11.58) *p* = 0.958	3 (6.52) *p* = 0.676	10 (14.71) *p* = 0.208	2 (9.09) *p* = 0.868	26 (11.26) *p* = 0.425	339 (9.43)
Mechanical lead damage (electrical failure)	42 (44.21) *p* < 0.001	18 (39.13) *p* = 0.076	20 (29.41) *p* = 0.676	4 (18.18) *p* = 0.529	84 (36.36) *p* = 0.001	949 (26.41)
Lead dysfunction (exit/entry block, dislodgement, perforation, extracardiac pacing)	11 (11.58) *p* = 0.013	8 (17.39) *p* = 0.474	13 (19.12) *p* = 0.548	3 (13.64) *p* = 0.434	35 (15.15) *p* = 0.008	825 (22.96)
Change of pacing mode/upgrading, downgrading	5 (5.26) *p* = 0.751	0 (0.00) *p* = 0.132	2 (2.94) *p* = 0.334	0 (0.00) *p* = 0.414	7 (3.03) *p* = 0.043	238 (6.62)
Other non-infectious indications *	12 (12.63) *p* = 0.902	5 (10.87) *p* = 0.876	9 (13.24) *p* = 0.950	6 (27.27) *p* = 0.087	32 (13.85) *p* = 0.698	458 (12.74)

TLE—transvenous lead extraction, M–W U test—Mann–Whitney U test, LVEF—left ventricular ejection fraction, NYHA FC—New York Heart Association functional class. * Abandoned lead/prevention of abandonment (atrial fibrillation, multiple leads), threatening/potentially threatening lead (loops, free end, left heart, lead-derived tricuspid valve defect), other (indications for magnetic resonance imaging, cancer, painful pocket, loss of indications for pacing/implantable cardioverter-defibrillator), and re-established venous access (symptomatic occlusion, superior vena cava syndrome, lead replacement/upgrading).

**Table 2:** System-, pacing history-, and procedure-related risk factors for major complications and procedure complexity.

System-Related Risk Factors for TLE Complexity and Major Complications	Lead Break with a Long Fragment	Lead Break with a Short Fragment	Lead Break of the Tip of the Lead Only	Lead Break with Loss of a Free-Floating Fragment	All Extractions, with Lead Break	All Extractions, without Lead Break
	Mean ± SD N (%) M–W U test, Chi^2^ test	Mean ± SD N (%) M–W U test, Chi^2^ test	Mean ± SD N (%) M–W U test, Chi^2^ test	Mean ± SD N (%) M–W U test, Chi^2^ test	Mean ± SD N (%) M–W U test, Chi^2^ test	Mean ± SD N (%)
Group number/number of patients (%)	1/95 (2.48)	2/46 (1.20)	3/68 (1.78)	4/22 (0.57)	5/231 (6.04)	6/3594 (93.96)
System and history of pacing						
Oldest lead before TLE (months)	188.8 ± 86.14 *p* < 0.001	177.7 ± 85.46 *p* < 0.001	151.7 ± 90.68 *p* < 0.001	191.7 ± 106.0 *p* < 0.001	176.0 ± 90.26 *p* < 0.001	97.66 ± 72.71
Global lead dwell time (years) before TLE	29.86 ± 17.69 *p* < 0.001	28.36 ± 13.37 *p* < 0.001	22.76 ± 13.00 *p* < 0.001	35.51 ± 21.89 *p* < 0.001	28.01 ± 16.46 *p* < 0.001	14.62 ± 12.28
Abandoned leads before TLE	27 (28.42) *p* < 0.001	10 (21.74) *p* = 0.008	14 (20.59) *p* = 0.004	12 (54.55) *p* < 0.001	63 (27.27) *p* < 0.001	354 (9.85)
Unnecessary (large) lead loop in the heart	10 (10.53) *p* = 0.005	6 (13.04) *p* = 0.005	3 (4.41) *p* = 1.000	7 (31.82) *p* < 0.001	26 (11.26) *p* < 0.001	158 (4.40)
Number of leads in the heart before TLE	2.16 ± 0.84 *p* = 0.004	2.20 ± 0.62 *p* = 0.005	2.10 ± 0.79 *p* = 0.071	2.68 ± 1.21 *p* = 0.027	2.20 ± 0.84 *p* < 0.001	1.94 ± 0.73
≥4 leads in the heart before TLE	8 (8.42) *p* < 0.001	0 (0.00) *p* = 0.260	5 (7.35) *p* < 0.001	7 (31.82) *p* < 0.001	20 (8.66) *p* < 0.001	98 (2.73)
Leads on both sides of the chest before TLE	7 (7.37) *p* = 0.003	0 (0.00) *p* = 0.280	6 (8.82) *p* < 0.001	5 (22.73) *p* < 0.001	18 (7.79) *p* < 0.001	89 (2.48)
Number of procedures before lead extraction	2.90 ± 1.67 *p* < 0.001	2.54 ± 1.24 *p* < 0.001	2.45 ± 1.41 *p* < 0.001	2.76 ± 1.14 *p* < 0.001	2.68 ± 1.47 *p* < 0.001	1.81 ± 1.03
Two or more CIED procedures before TLE	84 (83.42) *p* < 0.001	37 (80.43) *p* < 0.001	51 (75.00) *p* = 0.001	19 (86.36) *p* = 0.001	191 (82.68) *p* = 0.001	1834 (51.03)
Risk scores for prediction of major complications or increased procedure complexity.						
SAFeTY-TLE score (points)	10.75 ± 4.55 *p* < 0.001	9.62 ± 4.11 *p* < 0.001	6.36 ± 3.82 *p* < 0.001	10.07 ± 5.03 *p* < 0.001	9.75 ± 4.59 *p* < 0.001	5.67 ± 4.07
SAFeTY-TLE score (%)	5.80 ± 8.89 *p* < 0.001	3.69 ± 3.00 *p* < 0.001	3.36 ± 4.75 *p* < 0.001	3.89 ± 3.31 *p* < 0.001	4.48 ± 6.64 *p* < 0.001	1.54 ± 2.43
EROS score (points)	2.15 ± 0.92 *p* < 0.001	2.11 ± 0.82 *p* < 0.001	1.81 ± 0.87 *p* < 0.014	2.27 ± 0.83 *p* < 0.001	2.05 ± 0.89 *p* < 0.001	1.50 ± 0.71
MB score (points)	3.53 ± 0.68 *p* < 0.001	3.50 ± 0.78 *p* < 0.001	3.32 ± 0.89 *p* < 0.001	3.46 ± 0.91 *p* < 0.001	3.46 ± 0.79 *p* < 0.001	2.53 ± 1.25
LED index (points)	17.37 ± 7.11 *p* < 0.001	16.46 ± 7.08 *p* < 0.001	13.52 ± 7.46 *p* < 0.001	18.32 ± 9.04 *p* < 0.001	16.44 ± 7.48 *p* < 0.001	9.65 ± 6.10
Advanced TLE (Mazzone) scale (points)	2.53 ± 0.70 *p* < 0.001	2.50 ± 0.69 *p* = 0.006	2.49 ± 0.70 *p* = 0.001	2.41 ± 0.80 *p* = 0.154	2.50 ± 0.70 *p* < 0.001	2.12 ± 0.93
LECOM score (points)	12.97 ± 4.20 *p* < 0.001	12.45 ± 3.50 *p* < 0.001	11.20 ± 4.07 *p* < 0.001	14.18 ± 5.32 *p* < 0.001	12.36 ± 4.23 *p* < 0.001	7.94 ± 3.96
LECOM score (%)	41.81 ± 22.15 *p* < 0.001	39.76 ± 20.58 *p* < 0.001	33.07 ± 21.00 *p* < 0.001	50.34 ± 29.69 *p* < 0.001	39.64 ± 22.76 *p* < 0.001	19.08 ± 17.43
LECOM score (patients with very high expected complexity of the procedure)	79 (83.16) *p* < 0.001	33 (71.54) *p* < 0.001	45 (66.18) *p* < 0.001	18 (81.83) *p* < 0.001	175 (76.76) *p* < 0.001	1135 (31.58)
Potential procedure-related risk factors for major complications and increased procedure complexity.						
Number of extracted leads per patient	2.01 ± 0.88 *p* < 0.001	1.96 ± 0.70 *p* < 0.001	1.87 ± 0.69 *p* = 0.002	2.50 ± 1.10 *p* < 0.004	2.00 ± 0.82 *p* < 0.001	1.63 ± 0.72
Extraction of abandoned lead(s) (any)	26 (27.37) *p* < 0.001	10 (21.74) *p* = 0.003	13 (19.12) *p* = 0.004	11 (50.00) *p* < 0.001	60 (25.97) *p* < 0.001	322 (8.96)
Extraction of old model UP leads (excluding LV leads)	29 (30.53) *p* < 0.001	9 (19.57) *p* < 0.001	10 (14.71) *p* = 0.002	8 (36.36) *p* < 0.001	56 (24.24) *p* < 0.001	210 (5.84)
Extraction of passive fixation leads (excluding LV leads)	88 (92.63) *p* < 0.001	45 (97.83) *p* < 0.001	62 (91.18) *p* < 0.001	20 (90.91) *p* = 0.001	215 (93.07) *p* < 0.001	2008 (55.87)
Extraction of VDD leads	7 (7.37) *p* = 0.005	1 (2.17) *p* = 0.860	1 (1.47) *p* = 0.564	1 (4.55) *p* = 0.565	10 (4.33) *p* = 0.113	93 (2.59)
Extraction of leads with redundant loop in the heart	11 (11.58) *p* = 0.002	6 (13.04) *p* = 0.007	2 (2.94) *p* = 0.518	8 (36.36) *p* < 0.001	32 (13.85) *p* < 0.001	165 (4.59)
Oldest lead extracted per patient (months)	185.3 ± 83.85 *p* < 0.001	176.7 ± 85.69 *p* < 0.001	152.0 ± 90.68 *p* < 0.001	191.7 ± 106.04 *p* < 0.001	174.3 ± 89.25 *p* < 0.001	95.90 ± 71.68
Sum of dwell times of extracted leads (years)	28.46 ± 17.41 *p* < 0.001	26.13 ± 14.48 *p* < 0.001	21.29 ± 13.15 *p* < 0.001	35.51 ± 21.89 *p* < 0.001	26.56 ± 16.62 *p* < 0.001	12.96 ± 11.95

TLE—transvenous lead extraction; M–W U test—Mann–Whitney U test; CIED—cardiac implantable electronic device; SAFeTY-TLE score (prediction of major complications); EROS—ELECTRa Registry Outcome Score; MB—MB score; Advanced TLE Mazzone score—Mazzone score (need for advanced TLE techniques); LED—LED index, LECOM—Lead Extraction COMplexity score (prediction of TLE complexity); UP—unipolar; LV—left ventricle; VDD—dual chamber pacemaker (atrial sensing, ventricular sensing/pacing) with single ventricular lead.

**Table 3:** Procedure complexity in patients with lead breaks as compared to the control group.

Procedure Complexity	Lead Break with a Long Fragment	Lead Break with a Short Fragment	Lead Break of the Tip of the Lead Only	Lead Break with Loss of a Free-Floating Fragment	All Extractions, with Lead Break	All Extractions, without Lead Break
	Mean ± SD N (%) M–W U test, Chi^2^ test	Mean ± SD N (%) M–W U test, Chi^2^ test	Mean ± SD N (%) M–W U test, Chi^2^ test	Mean ± SD N (%) M–W U test, Chi^2^ test	Mean ± SD N (%) M–W U test, Chi^2^ test	Mean ± SD N (%)
Group number/number of patients (%)	1/95 (2.48)	2/46 (1.20)	3/68 (1.78)	4/22 (0.57)	5/231 (6.04)	6/3594 (93.96)
TLE complexity and outcomes						
Extraction time (sheath-to-sheath) (minutes)	69.80 ± 49.46 *p* < 0.001	27.20 ± 14.47 *p* < 0.001	24.74 ± 28.10 *p* < 0.001	92.73 ± 77.15 *p* < 0.001	50.23 ± 49.44 *p* < 0.001	12.77 ± 18.01
* Average time of single lead extraction (minutes)	38.75 ± 31.64 *p* < 0.001	15.70 ± 9.51 *p* < 0.001	12.83 ± 12.76 *p* < 0.001	44.09 ± 50.76 *p* < 0.001	27.04 ± 29.65 *p* < 0.001	7.75 ± 9.55
Overall number of technical problems in the group of patients	95 (100.0) *p* < 0.001	32 (69.57) *p* < 0.001	26 (38.24) *p* < 0.001	22 (100.0) *p* < 0.001	175 (75.76) *p* < 0.001	552 (15.36)
Number of technical problems per patient	2.21 ± 1.02 *p* < 0.001	1.53 ± 0.62 *p* < 0.001	1.54 ± 0.71 *p* < 0.001	2.73 ± 1.03 *p* < 0.001	2.05 ± 0.99 *p* < 0.001	1.24 ± 0.50
Two or more technical problems	69 (72.63) *p* = 0.001	15 (32.61) *p* = 0.001	12 (17.65) *p* = 0.001	22 (100.0) *p* = 0.001	118 (51.08) *p* = 0.001	115 (3.20)
Utilitization of additional tools and techniques						
Evolution (old and new) or TightRail	15 (15.79) *p* < 0.001	4 (8.70) *p* < 0.001	6 (8.82) *p* < 0.001	4 (18.18) *p* < 0.001	29 (12.55) *p* < 0.001	29 (0.81)
Metal sheaths	29 (30.53) *p* < 0.001	9 (19.57) *p* < 0.001	12 (17.65) *p* = 0.001	5 (22.73) *p* = 0.006	55 (23.81) *p* < 0.001	261 (7.26)
Lasso catheters/snares, basket catheters	91 (95.79) *p* < 0.001	12 (26.09) *p* < 0.001	4 (5.88) *p* = 0.014	22 (100.0) *p* < 0.001	129 (55.84) *p* < 0.001	65 (1.81)
Need to use an alternative approach	36 (37.89) *p* < 0.001	2 (4.35) *p* = 0.281	5 (7.35) *p* = 0.003	10 (45.45) *p* < 0.001	53 (22.94) *p* < 0.001	74 (2.06)
Procedure difficulty score						
** Retrospective TLE combined difficulty score (points)	3.58 ± 0.79 *p* < 0.001	1.98 ± 1.34 *p* < 0.001	1.04 ± 1.43 *p* < 0.001	3.82 ± 0.73 *p* < 0.001	2.54 ± 1.61 *p* < 0.001	0.43 ± 0.95
Retrospective TLE combined difficulty score of two or more points	95 (100.0) *p* < 0.001	33 (71.74) *p* < 0.001	23 (33.82) *p* < 0.001	22 (100.0) *p* < 0.001	173 (74.89) *p* < 0.001	556 (15.47)

TLE—transvenous lead extraction, M–W U test—Mann-Whitney U test, * (sheath-to-sheath/number of extracted leads), ** (dilatation time and the use of second line tools, advanced tools, and advanced techniques).

**Table 4:** Procedure complications and long-term outcomes.

Procedure Complications and Long-Term Outcomes	Lead Break with a Long Fragment	Lead Break with a Short Fragment	Lead Break of the Tip of the Lead Only	Lead Break with Loss of a Free-Floating Fragment	All Extractions, with Lead Break	All Extractions, without Lead Break
	N (%) Chi^2^ test	N (%) Chi^2^ test	N (%) Chi^2^ test	N (%) Chi^2^ test	N (%) Chi^2^ test	N (%)
Group number/number of patients (%)	1/95 (2.48)	2/46 (1.20)	3/68 (1.78)	4/22 (0.57)	5/231 (6.04)	6/3594 (93.96)
TLE efficacy and complications						
Major complications (any)	7 (7.37) *p* < 0.001	7 (15.22) *p* < 0.001	5 (7.35) *p* < 0.001	4 (18.18) *p* < 0.001	23 (9.96) *p* < 0.001	55 (1.53)
Hemopericardium	4 (4.21) *p* = 0.002	5 (10.87) *p* < 0.001	3 (4.41) *p* = 0.004	1 (4.55) *p* = 0.080	13 (5.63) *p* < 0.001	33 (0.92)
Hemothorax	1 (1.05) *p* = 0.014	0 (0.00) *p* = 0.821	0 (0.00) *p* = 0.783	0 (0.00) *p* = 0.877	1 (0.43) *p* = 0.190	4 (0.11)
Tricuspid valve damage during TLE (severe)	2 (2.11) *p* = 0.017	1 (2.17) *p* = 0.074	2 (2.94) *p* = 0.002	3 (13.64) *p* < 0.001	8 (3.46) *p* < 0.001	15 (0.42)
Rescue cardiac surgery	4 (4.21) *p* = 0.001	4 (8.70) *p* < 0.001	2 (2.94) *p* = 0.057	1 (4.55) *p* = 0.054	11 (4.76) *p* < 0.001	29 (0.81)
Death, procedure-related (intra-,post-procedural)	0 (0.00) *p* = 0.691	0 (0.00) *p* = 0.783	0 (0.00) *p* = 0.736	0 (0.00) *p* = 0.847	0 (0.00) *p* = 0.534	6 (0.17)
Clinical success	84 (88.42) *p* < 0.001	34 (73.91) *p* < 0.001	43 (63.24) *p* < 0.001	19 (86.36) *p* < 0.001	180 (77.92) *p* < 0.001	3567 (99.25)
Complete procedural success	58 (61.05) *p* < 0.001	6 (13.04) *p* < 0.001	1 (1.47) *p* < 0.001	12 (54.55) *p* < 0.001	77 (33.33) *p* < 0.001	3565 (99.19)
Long-term mortality after TLE						
Survivors	64 (67.37) *p* = 0.549	33 (71.74) *p* = 0.300	49 (72.06) *p* = 0.190	11 (50.00) *p* = 0.160	157 (67.97) *p* = 0.270	2314 (64.39)
One-year mortality	12 (12.63) *p* = 0.115	4 (8.70) *p* = 0.890	2 (2.94) *p* = 0.119	3 (13.64) *p* = 0.347	21 (9.09) *p* = 0.604	292 (8.13)
Overall follow-up mortality	31 (32.63) *p* = 0.549	13 (28.26) *p* = 0.300	19 (27.94) *p* = 0.190	11 (50.00) *p* = 0.160	74 (32.036) *p* = 0.270	1280 (35.62)

TLE—transvenous lead extraction.

**Table 5:** Management of lead break and its effectiveness.

Management of Lead Break	Lead Break with a Long Fragment	Lead Break with a Short Fragment	Lead Break of the Tip of the Lead Only	Lead Break with Loss of Broken Lead Fragment or Lead Tip	All Extractions, with Lead Break
	N (%)	N (%)	N (%)	N (%)	N (%)
Group number/number of patients (%)	1/95 (2.48)	2/46 (1.20)	3/68 (1.78)	4/22 (0.57)	5/231 (6.04)
Grasped remnant entirely removed	63 (66.32)	4 (8.70)	0 (0.00)	15 (68.18)	82 (35.50)
Shortening the lead remnant, with a retained fragment < 4 cm	26 (27.37)	1 (2.17)	0 (0.00)	3 (13.64)	30 (12.99)
Unsuccessful attempt at retrieval of the fragment	3 (3.16)	10 (21.74)	0 (0.00)	4 (18.18)	17 (7.36)
No attempt made to grasp the fragment (no chance)	0 (0.00)	29 (63.04)	68 (100.0)	0 (0.00)	97 (41.99)
Lead remnant removal during emergency or planned cardiac surgery	3 (3.16)	1 (2.17)	0 (0.00)	0 (0.00)	4 (1.73)
Procedure aborted due to major complications and ultimately, death of the patient	0 (0.00)	1 (2.17)	0 (0.00)	0 (0.00)	1 (0.43)

**Table 6:** Predictors of lead break: results of univariable and multivariable regression analysis.

	Univariable Regression Model			Multivariable Regression Model		
	OR	95% CI	*p*	OR	95% CI	*p*
Patient age during TLE (by 1 year)	0.977	0.970–0.984	<0.001			
Patient age at first system implantation (by 1 year)	0.965	0.959–0.971	<0.001	0.971	0.961–0.981	<0.001
Sum of all lead dwell times (by 1 year)	1.126	1.107–1.145	<0.001	1.018	1.005–1.031	0.006
Ischemic heart disease (*y/n*)	0.567	0.432–0.743	<0.001	1.125	0.803–1.577	0.494
LVEF (by 1% p)	1.027	1.017–1.038	<0.001	1.014	1.003–1.026	0.013
Charlson co-morbidity index (by 1 point)	0.912	0.875–0.951	<0.001	1.033	0.983–1.086	0.203
Non-infectious indications for TLE (*y/n*)	0.979	0.788–1.216	0.846			
Abandoned leads before TLE (*y/n)*	3.510	2.575–4.786	<0.001	1.107	0.712–1.720	0.652
Lead loops before TLE (*y/n*)	2.678	1.765–4.062	<0.001	1.347	0.856–2.120	0.198
Number of the leads in the heart before TLE (by 1)	1.526	1.296–1.798	<0.001	1.021	0.804–1.298	0.862
Leads on both sides of the chest (*y/n*)	1.020	0.980–1.062	0.335			
Number of CIED-related procedures before TLE (by 1)	1.692	1.537–1.863	<0.001	1.187	1.033–1.364	0.016
UP lead extraction (*y/n*)	4.627	3.377–6.341	<0.001	1.197	0.808–1.771	0.370
Extraction of passive fixation lead (*y/n*)	10.677	6.397–17.82	<0.001	6.354	3.679–10.97	<0.001
Sum of dwell times of all extracted leads (by 1 year)	1.056	1.048–1.065	<0.001			
Number of extracted leads (by 1)	1.784	1.526–2.085	<0.001			
Extraction of abandoned lead/leads (*y/n*)	3.671	2.681–5.026	<0.001			
Extraction of looped leads (*y/n*)	1.889	1.384–2.578	<0.001			
Dwell time of the oldest extracted lead (by 1 year)	1.129	1.110–1.148	<0.001			

TLE—transvenous lead extraction; LVEF—left ventricular ejection fraction; % *p*—percent point; CIED—cardiac implantable electronic device; UP—unipolar.

**Table 7:** Prognostic value of different scores predicting the risk or complexity of TLE in the evaluation of the probability of lead break; results of univariable and multivariable regression analysis.

	Univariable Regression Model			Multivariable Regression Model		
	OR	95%CI	*p*	OR	95%CI	*p*
SAFeTY-TLE (by 1% p)	1.198	1.160–1.237	<0.001	1.029	0.988–1.071	0.171
EROS (by 1 point)	2.396	2.036–2.819	<0.001	1.081	0.860–1.358	0.505
MB (by 1 point)	2.130	1.847–2.455	<0.001	1.128	0.895–1.422	0.307
LED (by 1 point)	1.129	1.110–1.148	<0.001	1.058	1.024–1.094	<0.001
Advanced TLE (by 1 point)	1.605	1.379–1.868	<0.001	1.128	0.898–1.416	0.300
LECOM (by 1% point)	1.041	1.035–1.047	<0.001	1.019	1.009–1.029	<0.001

SAFeTY-TLE score (predicting the risk of major complications); EROS—ELECTRa Registry Outcome Score; MB—MB score; LED—LED index; Advanced TLE (Mazzone) score—Mazzone score (need for use of advanced TLE techniques); LECOM—Lead Extraction COMplexity score (predicting extraction complexity).

## Data Availability

Readers can access the data supporting the conclusions of the study at www.usuwanieelektrod.pl.

## References

[1] Wilkoff B.L., Love C.J., Byrd C.L., Bongiorni M.G., Carrillo R.G., Crossley G.H., Epstein L.M., Friedman R.A., Kennergren C.E., Mitkowski P. (2009). Transvenous lead extraction: Heart Rhythm Society expert consensus on facilities, training, indications, and patient management: This document was endorsed by the American Heart Association (AHA). Heart Rhythm.

[2] Kusumoto F.M., Schoenfeld M.H., Wilkoff B., Berul C.I., Birgersdotter-Green U.M., Carrillo R., Cha Y.M., Clancy J., Deharo J.C., Ellenbogen K.A. (2017). 2017 HRS expert consensus statement on cardiovascular implantable electronic device lead management and extraction. Heart Rhythm.

[3] Bongiorni M.G., Burri H., Deharo J.C., Starck C., Kennergren C., Saghy L., Rao A., Tascini C., Lever N., Kutarski A. (2018). 2018 EHRA expert consensus statement on lead extraction: Recommendations on definitions endpoints research trial design data collection requirements for clinical scientific studies registries: Endorsed by APHRS/HRS/LAHRS. Europace.

[4] Smith H.J., Fearnot N.E., Byrd C.L., Wilkoff B.L., Love C.J., Sellers T.D. (1994). Five-years experience with intravascular lead extraction. U.S.Lead Extraction Database. Pacing Clin. Electrophysiol..

[5] Byrd C.L., Wilkoff B.L., Love C.J., Sellers T.D., Turk K.T., Reeves R., Young R., Crevey B., Kutalek S.P., Freedman R. (1999). Intravascular extraction of problematic or infected permanent pacemaker leads: 1994–1996. U.S. Extraction Database, MED Institute. Pacing Clin. Electrophysiol..

[6] Bongiorni M.G., Giannola G., Arena G., Soldati E., Bartoli C., Lapira F., Zucchelli G., Di Cori A. (2005). Pacing and implantable cardioverter-defibrillator transvenous lead extraction. Ital. Heart J..

[7] Gomes S., Cranney G., Bennett M., Li A., Giles R. (2014). Twenty-year experience of transvenous lead extraction at a single centre. Europace.

[8] Diemberger I., Migliore F., Biffi M., Cipriani A., Bertaglia E., Lorenzetti S., Massaro G., Tanzarella G., Boriani G. (2018). The “Subtle” connection between development of cardiac implantable electrical device infection and survival after complete system removal: An observational prospective multicenter study. Int. J. Cardiol..

[9] Kennergren C., Bucknall C.A., Butter C., Charles R., Fuhrer J., Grosfeld M., Tavernier R., Morgado T.B., Mortensen P., Paul V. (2007). Laser-assisted lead extraction: The European experience. Europace.

[10] Roux J.F., Pagé P., Dubuc M., Thibault B., Guerra P.G., Macle L., Roy D., Talajic M., Khairy P. (2007). Laser lead extraction: Predictors of success and complications. Pacing Clin. Electrophysiol..

[11] Wazni O., Epstein L.M., Carrillo R.G., Love C., Adler S.W., Riggio D.W., Karim S.S., Bashir J., Greenspon A.J., DiMarco J.P. (2010). Lead extraction in the contemporary setting: The LExICon study: An observational retrospective study of consecutive laser lead extractions. J. Am. Coll. Cardiol..

[12] Brunner M.P., Cronin E.M., Duarte V.E., Yu C., Tarakji K.G., Martin D.O., Callahan T., Cantillon D.J., Niebauer M.J., Saliba W.I. (2014). Clinical predictors of adverse patient outcomes in an experience of more than 5000 chronic endovascular pacemaker and defibrillator lead extractions. Heart Rhythm.

[13] Hussein A.A., Tarakji K.G., Martin D.O., Gadre A., Fraser T., Kim A., Brunner M.P., Barakat A.F., Saliba W.I., Kanj M. (2017). Cardiac Implantable Electronic Device Infections: Added Complexity and Suboptimal Outcomes with Previously Abandoned Leads. JACC Clin. Electrophysiol..

[14] Artus A., Mansourati J., Fatemi M., Pierre B., Schatz A., Badoz M., Laurent G., Guenancia C., Garnier F. (2022). Efficacy and safety of the new TightRail™ mechanical sheath for transvenous lead extraction: Results of a French multicenter study. J. Cardiovasc. Electrophysiol..

[15] Starck C.T., Gonzalez E., Al-Razzo O., Mazzone P., Delnoy P.P., Breitenstein A., Steffel J., Eulert-Grehn J., Lanmüller P., Melillo F. (2020). Results of the Patient-Related Outcomes of Mechanical lead Extraction Techniques (PROMET) study: A multicentre retrospective study on advanced mechanical lead extraction techniques. Europace.

[16] Yap S.C., Bhagwandien R.E., Theuns D.A.M.J., Yasar Y.E., de Heide J., Hoogendijk M.G., Kik C., Szili-Torok T. (2021). Efficacy and safety of transvenous lead extraction using a liberal combined superior and femoral approach. J. Interv. Card. Electrophysiol..

[17] Zhou X., Ze F., Li D., Wang L., Duan J., Yuan C., He J., Guo J., Li X. (2020). Transfemoral extraction of pacemaker and implantable cardioverter defibrillator leads using Needle’s Eye Snare: A single-center experience of more than 900 leads. Heart Vessels.

[18] Nof E., Bongiorni M.G., Auricchio A., Butter C., Dagres N., Deharo J.C., Rinaldi C.A., Maggioni A.P., Kutarski A., Kennergren C. (2019). Comparison of outcomes in infected cardiovascular implantable electronic devices between complete, partial, and failed lead removal: An ESC-EHRA-EORP ELECTRa (European Lead Extraction ConTrolled) registry. Europace.

[19] Calvagna G.M., Romeo P., Ceresa F., Valsecchi S. (2013). Transvenous retrieval of foreign objects lost during cardiac device implantation or revision: A 10-year experience. Pacing Clin. Electrophysiol..

[20] Kutarski A., Pietura R., Czajkowski M. (2012). Breakage of extracted leads: Another management option. Kardiol. Pol..

[21] Tanawuttiwat T., Cheng A., Rickard J., Chow G.V., Sciortino C.M., Brinker J. (2016). Successful extraction of right ventricular lead remnants using the FlexCath® steerable sheath. J. Interv. Card. Electrophysiol..

[22] Raatikainen M.J., Perälä J., Lahtinen J. (2009). Successful defibrillator lead remnant extraction from right ventricle using a steerable transseptal sheath and a basket retriever. Europace.

[23] Kutarski A., Chudzik M., Tomaszewski A., Pietura R., Oszczygiel A., Czajkowski M., Wranicz J.K. (2013). Difficult dual-stage transcutaneous multiple lead extraction with loss of external silicone tube of broken lead. Cardiol. J..

[24] Golzio P.G., Bongiorni M.G., Giuggia M., Vinci M., Gazzera C., Breatta A.D. (2007). Retrieval of pacemaker lead tip embolized into the distal pulmonary arterial bed during extraction procedure. Pacing Clin. Electrophysiol..

[25] Robinson T., Oliver J., Sheridan P., Sahu J., Bowes R. (2010). Fragmentation and embolization of pacemaker leads as a complication of lead extraction. Europace.

[26] Kim D., Baek Y.S., Lee M., Uhm J.S., Pak H.N., Lee M.H., Joung B. (2016). Remnant Pacemaker Lead Tips after Lead Extractions in Pacemaker Infections. Korean Circ. J..

[27] Jacheć W., Nowosielecka D., Ziaja B., Polewczyk A., Kutarski A. (2023). LECOM (Lead Extraction COMplexity): A New Scoring System for Predicting a Difficult Procedure. J. Clin. Med..

[28] Bontempi L., Vassanelli F., Cerini M., Inama L., Salghetti F., Giacopelli D., Gargaro A., Raweh A., Curnis A. (2017). Predicting the difficulty of a transvenous lead extraction procedure: Validation of the LED index. J. Cardiovasc. Electrophysiol..

[29] Bontempi L., Curnis A., Della Bella P., Cerini M., Radinovic A., Inama L., Melillo F., Salghetti F., Marzi A., Gargaro A. (2020). The MB score: A new risk stratification index to predict the need for advanced tools in lead extraction procedures. Europace.

[30] Mazzone P., Tsiachris D., Marzi A., Ciconte G., Paglino G., Sora N., Sala S., Vergara P., Gulletta S., Della Bella P. (2013). Predictors of advanced lead extraction based on a systematic stepwise approach: Results from a high volume center. Pacing Clin. Electrophysiol..

[31] Tułecki Ł., Jacheć W., Polewczyk A., Czajkowski M., Targońska S., Tomków K., Karpeta K., Nowosielecka D., Kutarski A. (2022). Assessment of the impact of organisational model of transvenous lead extraction on the effectiveness and safety of procedure: An observational study. BMJ Open.

[32] Jacheć W., Polewczyk A., Polewczyk M., Tomasik A., Kutarski A. (2020). Transvenous Lead Extraction SAFeTY Score for Risk Stratification and Proper Patient Selection for Removal Procedures Using Mechanical Tools. J. Clin. Med..

[33] Sidhu B.S., Ayis S., Gould J., Elliott M.K., Mehta V., Kennergren C., Butter C., Deharo J.C., Kutarski A., Maggioni A.P. (2021). Risk stratification of patients undergoing transvenous lead extraction with the ELECTRa Registry Outcome Score (EROS): An ESC EHRA EORP European lead extraction ConTRolled ELECTRa registry analysis. Europace.

[34] Morita J., Yamaji K., Nagashima M., Kondo Y., Sadohara Y., Hirokami J., Kuji R., Korai K., Fukunaga M., Hiroshima K. (2021). Predictors of lead break during transvenous lead extraction. J. Arrhythm..

[35] Gianni C., Elchouemi M., Helmy R., Spinetta L., La Fazia V.M., Pierucci N., Asfour I., Della Rocca D.G., Mohanty S., Bassiouny M.A. (2024). Safety and feasibility of same-day discharge following uncomplicated transvenous lead extraction. J. Cardiovasc. Electrophysiol..

